# Relationship between atypical language lateralization and cognition in pediatric patients with drug-resistant focal epilepsy: role of working memory

**DOI:** 10.3389/fneur.2026.1893275

**Published:** 2026-09-24

**Authors:** Johan S. Grisales-Cárdenas, Tania Jaramillo-Gil, Carlos R. Tolosa-Gaviria, Alejandro Herrera-Trujillo, Mauricio Palacios-Gómez, Christian A. Rojas-Cerón, Lina V. Becerra-Hernández, Efraín Buriticá-Ramírez, Carlos A. González-Acosta

**Affiliations:** 1 Centro de Estudios Cerebrales, Facultad de Salud, Universidad del Valle, Cali, Colombia; 2 Facultad de Psicología, Universidad Pontificia Bolivariana, Palmira, Colombia; 3 Clínica Imbanaco, Grupo Quirónsalud, Cali, Colombia; 4 Departamento de Ciencias Básicas de la Salud, Pontificia Universidad Javeriana, Cali, Colombia

**Keywords:** cognitive performance, drug-resistant focal epilepsy, functional magnetic resonance imaging, language lateralization, pediatric neurosurgery, working memory

## Abstract

**Introduction:**

Language lateralization is typically characterized by left-hemisphere dominance; however, atypical language organization occurs more frequently in pediatric patients with drug-resistant focal epilepsy (DRFE), likely reflecting adaptive neuroplastic mechanisms. Because surgical treatment is the standard of care for DRFE, understanding the relationship between language organization and cognitive functioning is essential to minimize postoperative deficits during ongoing neurodevelopment. This study aimed to characterize language lateralization using functional magnetic resonance imaging (fMRI) and examine its association with cognitive performance in pediatric patients undergoing presurgical evaluation.

**Methods:**

A retrospective study included 17 children aged 6–16 years with drug-resistant epilepsy evaluated between 2022 and 2025 at a tertiary epilepsy center. All patients underwent fMRI language paradigms targeting receptive, expressive, and mixed functions, alongside comprehensive neuropsychological assessment using the Wechsler Intelligence Scale for Children–Fourth Edition and the Evaluación Neuropsicológica Infantil battery. Language lateralization was quantified using a lateralization index and classified as typical left, bilateral atypical, or right atypical. Descriptive statistics, Spearman correlation analyses, and simple linear regression models were performed.

**Results:**

Seventeen patients were analyzed (mean age 11.65 years). Cognitive performance was globally below age expectations, with most participants classified within borderline or very low intellectual ranges. Atypical language lateralization was observed in approximately 80% of cases, predominantly right-hemisphere organization. Bilateral atypical lateralization showed a tendency toward lower cognitive scores. Negative correlations were identified between lateralization index and working memory and visuospatial measures, although these associations lost significance after correction for multiple comparisons. Regression analysis indicated that language organization explained part of the variance in working memory performance.

**Discussion:**

Atypical language organization is highly prevalent in pediatric DRFE and may represent adaptive cortical reorganization. These findings highlight the relevance of fMRI language mapping for presurgical planning and understanding neurocognitive outcomes in developing brain.

## 1 Introduction

Language lateralization, typically characterized by left-hemisphere dominance for both expressive (frontal) and receptive (temporoparietal) language functions, accounts for approximately 90–95% of cortical organization in healthy individuals (1–3). Deviations from this pattern, such as bilateral or right-hemisphere language representation, are uncommon in healthy populations but may occur more frequently in certain pathological conditions (4). Epilepsy, particularly focal epilepsy (FE), provides a relevant clinical model given its predominantly unilateral hemispheric involvement and its frequent association with indispensable language cortices (5, 6). Moreover, pediatric patients with FE constitute a key population for investigating atypical language lateralization (ALL), as they are undergoing active cognitive and cortical maturation, including the development of language networks (7–10). In this context, drug-resistant focal epilepsy (DRFE) emerges as a significant concern, because surgical intervention becomes the gold-standard treatment (11). This approach implies lesional procedures to achieve seizure freedom, with the potential risk of functional deficits that may include cognitive impairment and an impact on functional networks still undergoing maturation in pediatric patients with DRFE.

FEs are the predominant epilepsy type in childhood (12), and about 10 to 40% of pediatric epilepsies become drug-resistant (13–16). There are a high incidence and prevalence of drug-resistance epilepsies (DRE) in the first year of life, predominantly in low- and middle-income countries (17). Surgical intervention for FE involves delineating the epileptogenic zone (EZ), defined as the cortical sector whose removal offers the highest probability of seizure freedom with minimal functional retraction (18, 19). Pre-surgical assessment (Figure 1) aids in finding such a zone and, thus, reduces potential post-surgical cognitive impairments.

**Figure 1:**
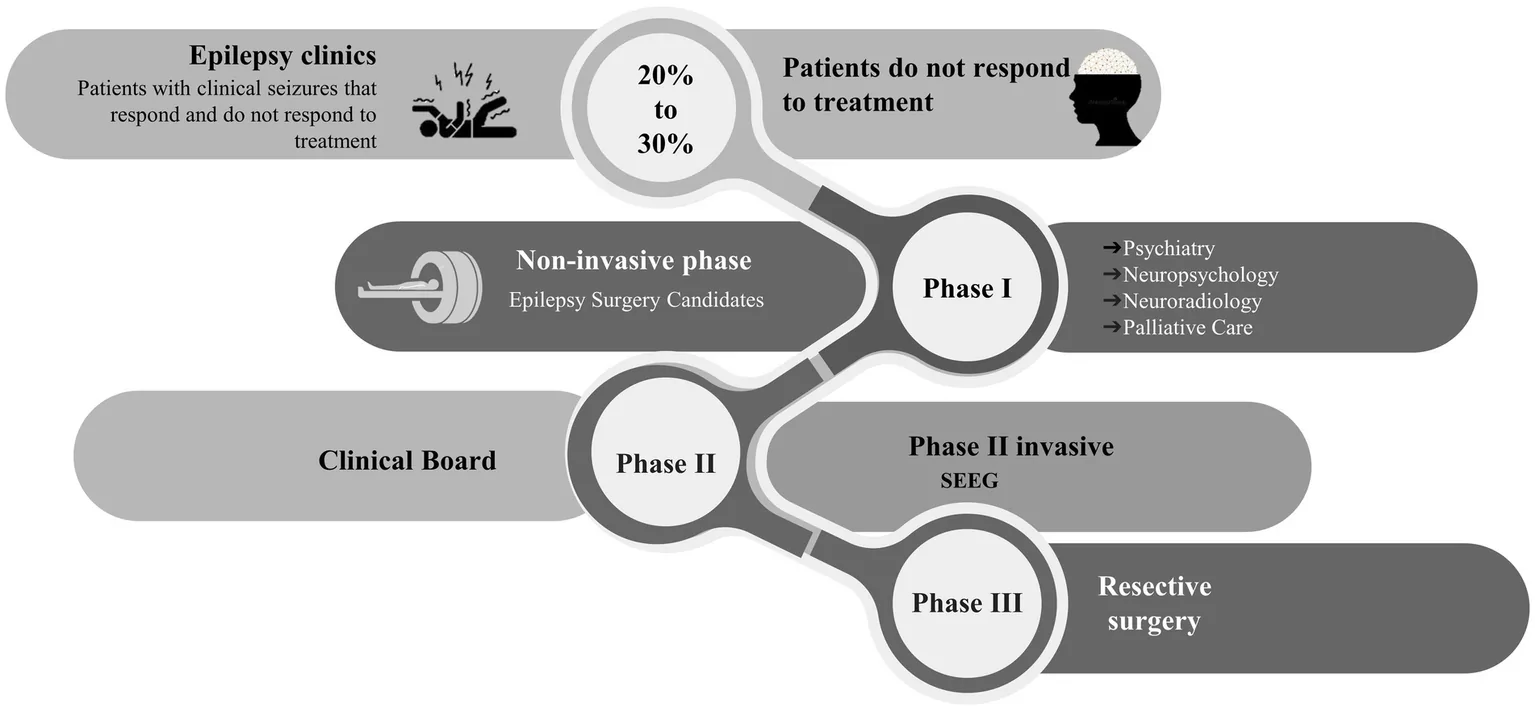
Clinical assessment protocol for epilepsy surgery candidates.

Given the ongoing cortical maturation processes during childhood and adolescence, including language development, reducing the risk for cognitive impairment is crucial; besides, the influence of early language organization on other cognitive domains remains poorly understood, particularly in the context of potential disruptions caused by conditions such as DRFE. Some studies suggest that pediatric DREs exert a pervasive impact on the development of emerging cortical functions, affecting attention, memory, and executive functions (20, 21). Moreover, intellectual skills appear to be compromised, particularly in younger children with drug-resistant epilepsy, with influencing factors such as illness duration, presence of interictal activity, frequency, and other relevant variables (13). In terms of language abilities, children with epilepsy frequently demonstrate deficits and often perform approximately one standard deviation below their neurotypical peers (22, 23).

Early surgical intervention in children with DRFE can improve the prognosis of cognitive development, provided that arrest resulting from the lesion of an eloquent region is avoided, particularly in memory and language regions (24). From this perspective, neuropsychological assessment through functional magnetic resonance imaging (fMRI) studies can be important in establishing the baseline cognitive profile, localizing the dysfunctional zone, and identifying eloquent regions (25).

Children with DRFE may exhibit MRI abnormalities constituting structural etiologies, such as developmental cortical malformations, neoplasms, and volumetric reduction of the parenchyma. Some changes may precede the onset of seizures or even be a consequence. In cases of DRFE involving eloquent areas of language, a reorganization of the underlying networks may occur at the intra- and inter-hemispheric levels, which is thought to lead to an ALL as a mechanism of neuronal plasticity in response to the lesion (26). Indeed, ALL has been reported to be increased in DRFE patients compared to healthy subjects (27).

When analyzing the relationship between language lateralization and cognitive performance, it is important to note that specific language impairments have been identified in pediatric epilepsy cases even when intellectual skills are within the average range (23). Furthermore, language deficits have been previously suggested to influence the development of other cognitive domains, such as executive functions and attention (28, 29). Interestingly, some studies have pointed out that this population would have a reduced risk for cognitive impairment following resective surgery if performed on the typical language-dominant left hemisphere (30).

The relationship between language lateralization and cognition is significant in the pediatric population. Reports suggest that ALL is more prevalent after early acquired lesions (4, 30). Additionally, while it has been observed that ALL is not linked with poorer performance on language tasks in healthy children (3), it’s not clear if and how cognitive dysfunction and increased ALL in pediatric epilepsy might be related. For instance, one study identified an association between ALL and better verbal memory performance (31). Also, ALL seems more frequent in FE than in a healthy population (32).

This study aims to characterize language lateralization using fMRI in pediatric patients with DRFE who have been identified as candidates for resective surgery. These results would then make it possible to establish the relationship between performance on cognitive assessments and language reorganization.

## 2 Materials and methods

### 2.1 Participants

A retrospective review was conducted of medical records from children (6–16 years) with DRFE admitted between 2022 and 2025 to a tertiary epilepsy center. This cross-sectional study included patients undergoing Phase I of the presurgical evaluation for epilepsy surgery (see Figure 1) consisting of non-invasive diagnostic studies, who had undergone functional MRI (fMRI) with a language paradigm and a comprehensive neuropsychological assessment. Complete multidisciplinary documentation was available for all participants. Patients with epileptic syndromes or generalized epilepsy were excluded (Figure 2). Data from all participants included, encompassing sociodemographic, clinical, cognitive and language lateralization index is included as Supplementary Table S1.

**Figure 2:**
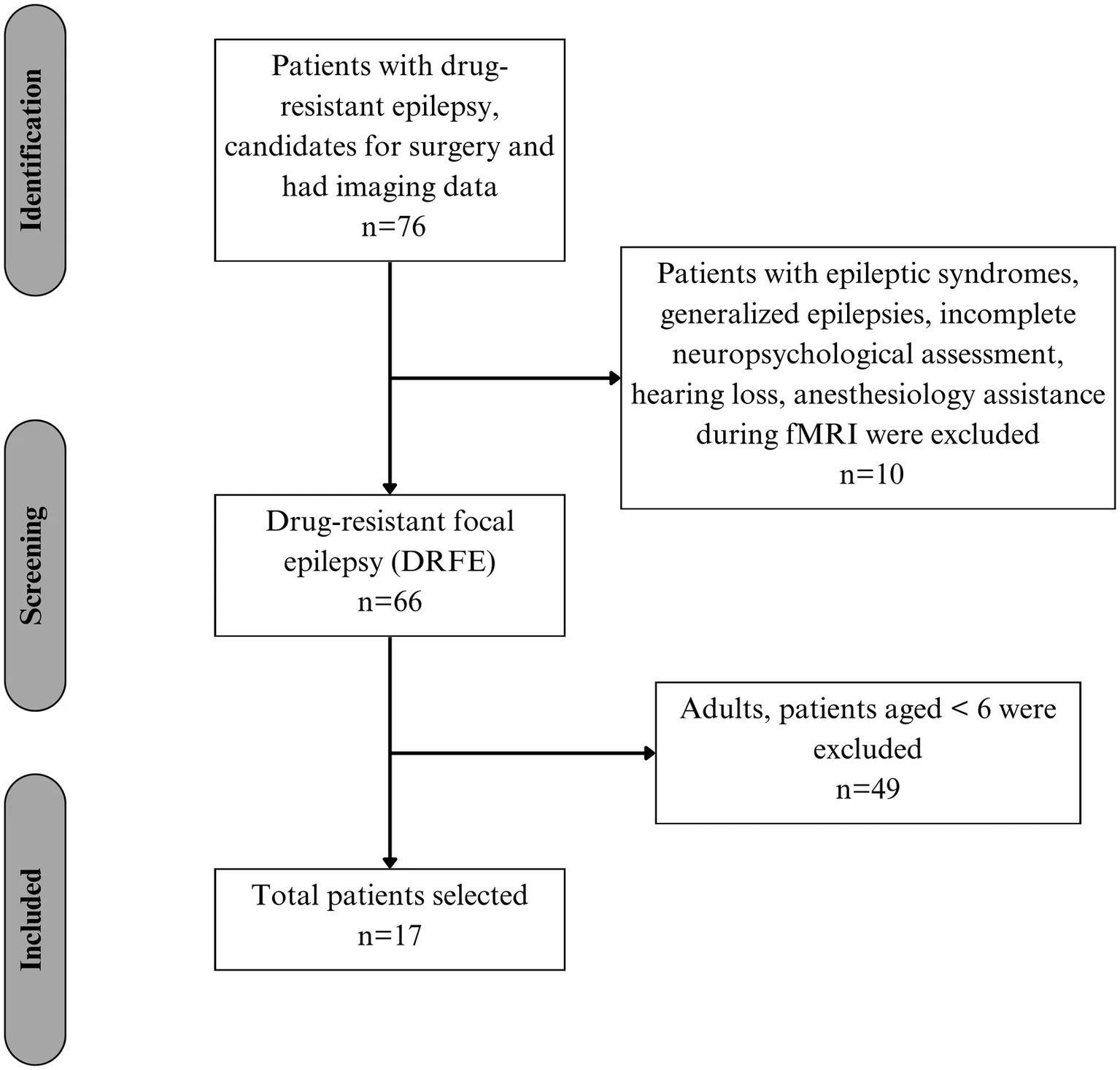
Sample selection flowchart.

### 2.2 Neuropsychological tests

Neuropsychological evaluation was conducted as part of the presurgical workup and included standardized measures of global intellectual functioning and domain-specific cognitive abilities. Intellectual functioning was assessed using the Wechsler Intelligence Scale for Children–Fourth Edition (WISC-IV) (33). The Full-Scale Intelligence Quotient (FSIQ) and index scores—Verbal Comprehension (VC), Perceptual Reasoning (PR), Working Memory (WM), and Processing Speed (PS)—were included in the analysis. Domain-specific cognitive functions were evaluated using the Evaluación Neuropsicológica Infantil (ENI) (34), specifically the attention, memory, language, and visuospatial subtests. The ENI is a standardized battery designed for spanish-speaking pediatric populations that allows deriving percentile scores, which the ENI manual defines as follows: >75 above average, 26–75 average, 11–25 below average, 3–10 low, ≤ extremely low.

### 2.3 fMRI

Magnetic resonance imaging was performed on a 3 T Siemens MAGNETOM Skyra scanner with a 20-channel head coil. As part of the presurgical epilepsy protocol, structural and functional sequences relevant to the present study were acquired. High-resolution structural images were obtained using a whole-brain 3D T1-weighted magnetization-prepared rapid gradient-echo (MPRAGE) sequence (voxel size = 0.94 × 0.94 × 0.94 mm; TR = 2,200 ms; TE = 2.5 ms; FA = 8°; FOV = 240 mm; 240 slices). Functional images were acquired using a gradient-echo echo-planar imaging (EPI) blood oxygen level–dependent (BOLD) sequence (TR = 3,790 ms; TE = 30 ms; FA = 90°; 36 slices; voxel size = 3 × 3 × 4.38 mm^3^; 60 volumes).

All images were anonymized and converted to NIfTI format using dcm2niix (35). Preprocessing and first-level analyses were performed using the Brain Extraction Tool (BET) (36) and FMRI Expert Analysis Tool (FEAT) (37) within the FSL software package (38). The preprocessing pipeline included motion correction using rigid-body transformations, slice-timing correction for interleaved acquisition, intensity normalization, spatial smoothing with a 7 mm full-width at half-maximum (FWHM) Gaussian kernel, and high-pass temporal filtering. Functional images were registered to skull-stripped structural images using 12 degrees of freedom and subsequently normalized to standard Montreal Neurological Institute (MNI) space using affine transformations (12 degrees of freedom).

Statistical parametric maps were generated by contrasting activation and rest blocks (39 s each), yielding voxel-wise z-statistics and cluster sizes. Statistical maps were thresholded using a cluster-forming threshold of *Z* > 3.1 and a cluster-level family-wise error-corrected significance threshold of *p* < 0.05, based on Gaussian Random Field theory. Activation maps were overlaid onto individual anatomical images to identify regions of interest and determine language lateralization patterns. Figure 3 illustrates representative examples of typical left-lateralized, bilateral atypical, and right-lateralized activation during language tasks.

**Figure 3:**
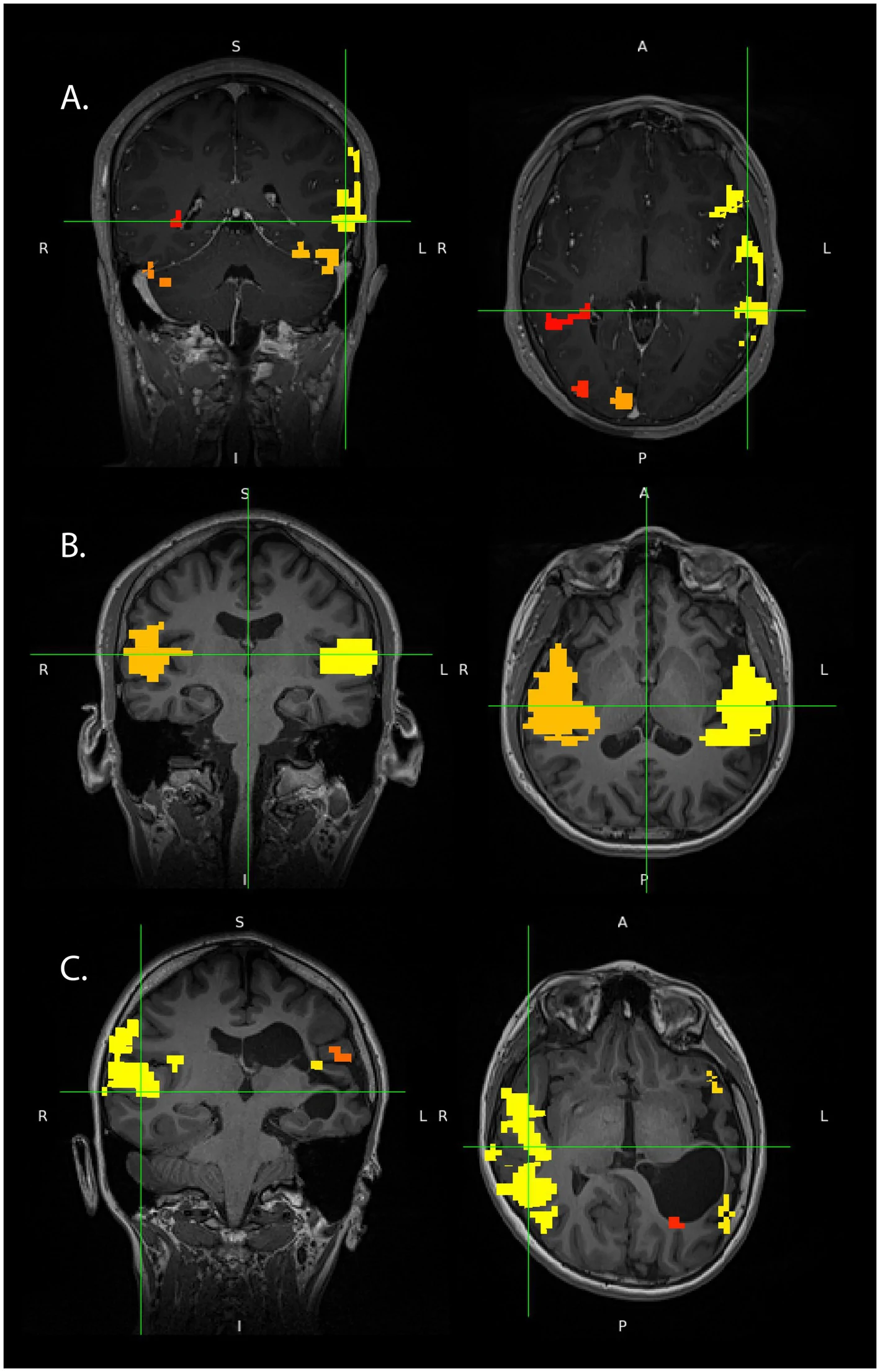
T1-weighted anatomical images of three of the patients with an fMRI-derived activation mask superimposed showing a **(A)**, typical, **(B)** bilateral atypical, and **(C)** right atypical brain activity during the performance of language tasks.

### 2.4 Language paradigms

The fMRI protocol included three language paradigms targeting predominantly receptive, expressive, and mixed (receptive–expressive) functions. Given sociodemographic and clinical factors, many patients had not achieved literacy or demonstrated limited reading performance. As auditory-verbal communication is typically the most developed and relatively preserved modality in this population—and the primary function prioritized for postoperative preservation—auditory-verbal stimuli were selected.

The receptive language task consisted of passive listening to a short, age-appropriate story. Participants were instructed to attend to the narrative and retain as much information as possible. The mixed task involved listening to simple but incomplete sentences, which participants were instructed to complete mentally. The expressive task required the covert repetition of semantically unrelated, high-frequency everyday words. To minimize motion-related artifacts, participants were instructed to perform all responses mentally.

### 2.5 Calculation of the lateralization index and classification by typicality

Language activation was quantified within predefined regions of interest and visually inspected using FSLeyes. For receptive language, regions of interest included the superior and middle temporal gyri, the temporoparietal junction, and the inferior parietal lobule, whereas expressive language analyses focused on the frontal operculum and the triangular portion of the inferior frontal gyrus. The lateralization index (LI) was calculated using the formula LI = 100 × (L − R)/(L + R), where L represents the number of suprathreshold voxels in the left hemisphere and R the number in the right hemisphere. In accordance with previous literature (39), language lateralization was classified as left-typical (LTL) when LI ≥ 20, bilateral atypical (BAL) when −20 < LI < 20, and right-atypical (RAL) when LI ≤ −20 (Figure 3).

### 2.6 Statistical analysis

Descriptive statistics were computed for the whole sample and for the LTL, BAL, and RAL groups across sociodemographic, clinical, cognitive, and lateralization variables. Given the sample size, both mean ± standard deviation and median with interquartile range were examined; as results were comparable, mean ± standard deviation was reported, except for seizure frequency, for which both measures are presented. Owing to the limited sample size, no between-group comparisons were performed.

Spearman’s rank correlation analyses were conducted among numerical variables, with particular focus on associations between the lateralization index (LI) and sociodemographic, clinical, and cognitive measures. To account for multiple testing, *p*-values were adjusted using Holm’s correction method. Associations between categorical variables and language lateralization were assessed using Fisher’s exact test.

Finally, numerical variables that showed significant correlations with LI were entered as dependent variables in simple linear regression models to evaluate the influence of cortical language organization on cognitive outcomes.

### 2.7 Ethics statement

The Clinica Imbanaco – Grupo QuironSalud institutional review board reviewed and approved this project, registered in Minute CEI - 9 dated August 27, 2021. Data were collected from clinical and paraclinical analysis in the context of their diagnosis and treatment. The information was anonymized and coded to prevent the exposure of patients’ IDs.

The study adhered to the principles outlined in the Declaration of Helsinki, as well as the Scientific, Technical, and Administrative Standards for Health Research established in Resolution No. 008430 of 1993 by the Ministry of Health, and the Standards for the Management of Clinical History established in Resolution No. 1995 of 1999 by the Ministry of Health of Colombia.

## 3 Results

### 3.1 Sociodemographic and clinical characteristics

Sociodemographic and clinical features of the sample are summarized in Table 1. Seventeen children were included in the final sample (7 female, 10 male), with ages ranging from 6 to 16 years old (*M* = 11.65, SD = 3.18). Regarding handedness, 13 patients (76.47%) were right-handed, two (11.76%) were left-handed, and two (11.76%) exhibited mixed handedness. The group had an average age of seizures onset of 4.64 years (3.40), and a mean duration of epilepsy of 7.01 years (3.37). The most frequent type of seizure was nonmotor to motor with awareness impairment (58.28%), and as for yearly ictal frequency, these ranged from 6 to 672 (*M* = 121.94, SD = 186.52, Med = 24, IQR = 15–96). Regarding the epileptogenic zone, 15 patients (88.24%) had a left hemisphere EZ, and two patients (11.76%) had a right hemisphere EZ.

**Table 1:** Sociodemographic and clinical features of the sample.

Variable	Value
Number of patients	17
Age	11.65 (3.18)
Sex, f/m	7/10
Education	
Complete/incomplete secondary school	9 (52.94%)
Complete/incomplete primary school	6 (35.29%)
Informal education	1 (5.88%)
None	1 (5.88%)
Handedness, r/l/m	13/2/2
Age of seizures onset	4.64 (3.40)
Epilepsy duration	7.01 (3.37)
Seizure type	
Motor	2 (11.76%)
Motor with awareness impairment	1 (5.88%)
Nonmotor with awareness impairment	2 (11.76%)
Nonmotor to motor with awareness impairment	10 (58.82%)
Nonmotor with awareness impairment to motor with awareness impairment	2 (11.76%)
Seizures frequency, year (mean, SD, median, IQR)	121.94, 186.52, 24 (15–96)
Number of antiseizure medications	
2	5 (29.41%)
3	9 (52.94%)
4	3 (17.65%)
Antiseizure medications^a^	
Carbamazepine	1 (7.69%)
Clobazam	5 (38.46%)
Levetiracetam	11 (84.62%)
Lacosamide	5 (38.46%)
Oxcarbazepine	5 (38.46%)
Topiramate	2 (15.38%)
Valproic acid	5 (38.46%)
Vigabatrin	2 (15.38%)
Type of lesion	
Mesial sclerosis	1 (5.88%)
Gliosis (perinatal lesion)	4 (23.53%)
Low-grade neoplasm	2 (11.76%)
Developmental cortical malformation	7 (41.18%)
None	3 (17.65%)
Hemisphere of epileptogenic zone, l/r	15/2

Values are presented either as proportions, frequency (%) or mean (standard deviation). ^a^This data was only available in the medical records of 13 of the participants, and as such, the percentages were calculated based on 13.

Fourteen patients had a structural etiology, in which the most frequent lesion was developmental cortical malformations (*n* = 7; 41.18%), followed by perinatal encephalopathy-associated gliosis (*n* = 4; 23.53%), low-grade neoplasms (*n* = 2; 11.76%), and one case of mesial sclerosis (*n* = 1; 5.88%). Additionally, three cases of non-lesional epilepsy were identified (*n* = 3; 17.65%), from which two had an infectious etiology (post-COVID-19) and the other had an unknown etiology. Regarding pharmacological scheme treatment, five patients (29.41%) received two antiseizure medications, nine (52.94%) received three, and three (17.65%) received four medications; notably, no patient was on a monotherapy scheme. Among the 13 patients for whom detailed information on the specific medications used was available, levetiracetam was the most frequently used antiseizure medication, present in 11 patients (84.62%), followed by oxcarbazepine, clobazam, lacosamide and valproic acid, each used in five patients (38.46%). Topiramate and vigabatrin were each used in two patients (15.38%), and carbamazepine was used in one patient (7.69%). Although education data is provided, caution must be taken when considering it, since it might confound analyses because some patients have been promoted in their schools without reaching the expected academic achievements. This happens especially when those schools do not have the resources to handle these children’s educational requirements.

### 3.2 Cognitive performance

Table 2 summarizes the cognitive test scores obtained by the patients. Overall, most of the performances in the ENI tasks were below age-expected norms (see ENI percentile scores in Supplementary Table S2). In the tasks measuring attention, figures and letters cancelation, 41 and 47% of the participants had percentile scores of 2 or less (extremely low), respectively, while 12 and 35% of the participants had percentile scores between 3 and 10 (low), respectively; finally, only in the figures cancelation test 6% (1 participant) of the participants had a percentile score between 11 and 25 (below average). The rest of the participants had an average (26–75) percentile score in these two tasks (41 and 18%, respectively).

**Table 2:** Cognitive test scores of the sample.

Cognitive measure	Mean score/Value
Full-scale IQ	65.88 (13.42)
Processing speed index	74.76 (14.86)
Verbal comprehension index	69 (15.78)
Perceptual reasoning index	75.65 (14.62)
Working memory index	69.18 (12.46)
IQ classification	
Very superior	0
Superior	0
Average high	0
Average	0
Average low	3 (17.65%)
Borderline	6 (35.29%)
Very low	8 (47.06%)
ENI	
Attention	
Figures	22.82 (13.28)
Letters	18.47 (12.17)
Backward digits	3.59 (1.84)
Memory	
Auditory list, coding	24.29 (8.79)
Auditory list, recall	5.82 (2.81)
Complex figure, recall	6.53 (3.66)
Visuospatial	
Complex figure, copy	9.47 (4.62)
Language	
Naming	10.41 (3.26)
Instructions	7.62 (1.41)
Word-picture matching	13.50 (3.09)

Values are presented as mean (standard deviation), except for IQ Classification whose values are presented as frequencies (%).

Backward digits span, although included as an attention test in the ENI battery, has classically been considered an executive function (working memory) task. In said task, 12% of the participants had extremely low percentile scores, 24% had low percentile scores and 6% had below average percentile scores, while the rest of them (59%) had average percentile scores. Visuospatial skills, assessed through the copy of a complex figure test, yielded 24% of the participants with extremely low percentile scores, 18% had low percentile scores and 6% had below average scores, with the rest of participants (53%) having average percentile scores.

Memory tests included auditory list coding and recall, as well as complex figure recall. 29, 41 and 24% of the participants had extremely low percentile scores in these tasks, respectively; 35, 24 and 18% had low percentile scores, respectively; and only 6% of the participants in both auditory list coding and complex figure recall had below average percentile scores. One participant (6%) reached an above average percentile score in the auditory list coding task. As for the rest of the participants (24, 35 and 53%, respectively), they all had average percentile scores.

Finally, language tasks included naming, instructions’ following and word-picture matching. 12, 35 and 18% of the participants had extremely low percentile scores in these tasks, respectively; 12, 12 and 6% had low percentile scores, respectively; and only 6% in the naming task and 12% in the instructions task had below average percentile scores. Interestingly, 18% of participants in the naming task had an above average percentile score. The rest of the participants (53, 41 and 76%, respectively) had average percentile scores.

Regarding the WISC-IV battery, IQ classifications included three patients classified as low average, six as borderline, and eight as very low. None of the participants fell within the average or higher ranges. Specifically, for the Processing Speed index the group had an average score of 74.76 (SD = 14.86), for the Verbal Comprehension index an average score of 69 (SD = 15.78), for Perceptual Reasoning index an average of 75.65 (SD = 14.62), for the Working Memory index an average of 69.18 (SD = 12.46), and for the Full-Scale IQ an average of 65.88 (13.42).

### 3.3 Language lateralization

Figure 4 illustrates the cortical language organization of patients as measured by the LI. According to the predefined cutoff values described in the Methods section, 4 patients exhibited typical left language organization (*M* = 38.72, SD = 18.15), 4 showed bilateral atypical organization (*M* = 0.39, SD = 10.64), and 9 demonstrated right atypical organization (*M* = −44.09, SD = 12.31).

**Figure 4:**
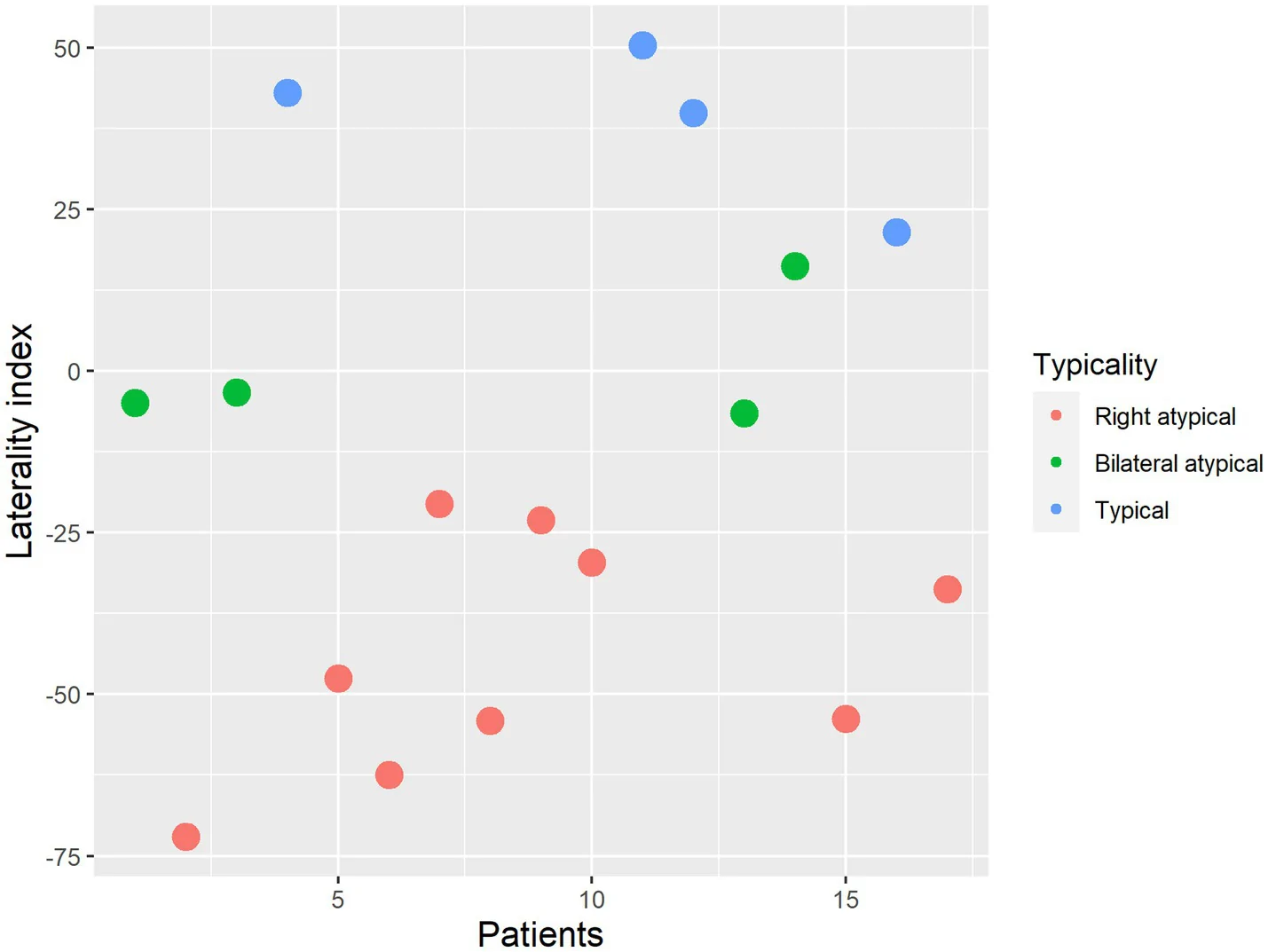
Patients’ language lateralization typicality based on the fMRI-derived laterality index.

### 3.4 Sociodemographic and clinical features by language lateralization pattern

A summary of the sociodemographic and clinical characteristics based on language LI is presented in Table 3. Given the limited sample size in each subgroup, analyses were restricted to descriptive statistics, and no inferential comparisons were conducted. Males were more common in the RAL and BAL groups (3F/6M and 1F/3M, respectively), while females were common in the LTL group (3F/1M). Mean age was 12.33 years (SD = 3.28) in the RAL group, 11.50 years (SD = 3.11) in the BAL group, and 10.25 years (SD = 3.40) in the LTL group. Regarding handedness, two patients were left-handed (both RAL), and two exhibited mixed handedness (1 BAL and 1 LTL).

**Table 3:** Sociodemographic and clinical features by language lateralization LI.

Variable	RAL *N* = 9	BAL *N* = 4	LTL *N* = 4
Age	12.33 (3.28)	11.50 (3.11)	10.25 (3.40)
Sex, females/males	3/6	1/3	3/1
Education			
Complete/incomplete secondary school	5	2	2
Complete/incomplete primary school	4	0	2
Informal education	0	1	0
None	0	1	0
Handedness, r/l/m	7/2/0	3/0/1	3/0/1
Age of seizures onset, years	5.22 (2.99)	5.25 (5.19)	2.70 (2.12)
Epilepsy duration, years	7.11 (3.59)	6.25 (3.30)	7.55 (3.78)
Seizure type			
Motor	1	1	0
Motor with awareness impairment	0	0	1
Nonmotor with awareness impairment	2	0	0
Nonmotor to motor with awareness impairment	6	2	2
Nonmotor with awareness impairment to motor with awareness impairment	0	1	1
Seizures frequency, year (mean, SD, median, IQR)	136.33, 226.37, 24 (12–96)	114, 148.43, 48 (42–120)	97.50, 159.23, 24 (19.5–102)
Number of antiseizure medications, 2/3/4	4/4/1	0/3/1	1/2/1
Antiseizure medications^a^			
Carbamazepine	0	0	1
Clobazam	4	1	0
Levetiracetam	6	3	2
Lacosamide	3	0	2
Oxcarbazepine	2	2	1
Topiramate	0	1	1
Valproic acid	3	1	1
Vigabatrin	1	0	1
Type of lesion			
Mesial sclerosis	0	1	0
Gliosis	3	0	1
Low-grade neoplasm	2	0	0
Developmental cortical malformation	3	1	3
None	1	2	0
Hemisphere of epileptogenic zone, l/r	8/1	4/0	3/1

Values are presented either as proportions, frequencies or mean (standard deviation). r, right; l, left; m, mixed. ^a^This data was only available in the medical records of 13 of the participants; as for the other 4 participants, 2 belonged to the RAL group, 1 to BAL and 1 to LTL.

The mean age at seizure onset in atypical patients, both RAL and BAL, was seemingly higher (*M* = 5.22, SD = 2.99; *M* = 5.25, SD = 5.19, respectively), than in the LTL patients (*M* = 2.70, SD = 2.12). The duration of epilepsy ranged from 1 to 12 years across groups, with mean durations of 7.11 years (SD = 3.59) in RAL, 6.25 years (SD = 3.30) in BAL, and 7.55 years (SD = 3.78) in LTL. Regarding ictal semiology, nonmotor-to-motor seizures with impaired awareness were the most frequent type in all three groups (RAL = 6; BAL = 2; LTL = 2). Yearly seizure frequency showed a markedly non-normal distribution with substantial interindividual variability, limiting meaningful descriptions. For example, although the RAL group appeared to present a higher mean annual frequency (*M* = 136.33), the median was considerably lower (Median = 24), reflecting dispersion in the data (SD = 226.37; IQR = 12–96).

Regarding lesional findings, developmental cortical malformations (DCM) were the most frequent lesions across groups (RAL = 3, BAL = 1, LTL = 3). Other etiologies included gliosis (RAL = 3, BAL = 0, LTL = 1), low-grade neoplasms (RAL = 2, BAL = 0, LTL = 0), and mesial temporal sclerosis (RAL = 0, BAL = 1, LTL = 0). Three cases of non-lesional epilepsy were also identified (RAL = 1, BAL = 2, LTL = 0). Only two patients presented with a right-sided EZ (RAL = 1, LTL = 1).

Finally, as for the antiseizure schemes, participants receiving a scheme of three antiseizure medications was rather common across groups (RAL = 4, BAL = 4, LTL = 2), followed by schemes of two antiseizure medications (RAL = 4, BAL = 0, LTL = 1) and schemes of four antiseizure medications (RAL = 1, BAL = 1, LTL = 1). Specifically, levetiracetam was the most common medication across groups (RAL = 6, BAL = 3, LTL = 2), while some other medications were not comparably frequent across groups, for example, lacosamide (RAL = 3, BAL = 0, LTL = 2), oxcarbazepine (RAL = 2, BAL = 2, LTL = 1), clobazam (RAL = 4, BAL = 1, LTL = 0), and valproic acid (RAL = 3, BAL = 1, LTL = 1). More uncommon were the likes of vigabatrin (RAL = 1, BAL = 0, LTL = 1), topiramate (RAL = 0, BAL = 1, LTL = 1) and carbamazepine (RAL = 0, BAL = 0, LTL = 1).

### 3.5 Cognitive performance by language lateralization pattern

Cognitive results organized by language lateralization are presented in Table 4. Some regularities were observed, whereby BAL participants had lower mean scores than the other two groups on most tests, with some exceptions. In two of the three ENI language subtests (naming and word–picture matching), as well as in the ENI backward digit span, the mean scores were organized in a different way: patients with more typical lateralization showed lower performance, whereas those with more atypical organization tended to perform better on these language- and working memory–related measures. Additionally, LTL patients demonstrated greater variability in cognitive performance compared to the other groups.

**Table 4:** Cognitive test scores by language lateralization.

Cognitive measure	RAL *N* = 9	BAL *N* = 4	LTL *N* = 4
WISC-IV			
Full-scale IQ	71.56 (11.30)	51.25 (8.06)	67.75 (12.95)
Processing speed index	79.78 (11.45)	59.75 (7.50)	78.50 (19.42)
Verbal comprehension index	74.11 (15.27)	55 (5.89)	71.50 (17.99)
Perceptual reasoning index	78.44 (13.68)	63.50 (11.36)	81.50 (15.72)
Working memory index	76.78 (10.33)	60.50 (7.55)	60.75 (10.87)
IQ classification			
Very superior	0	0	0
Superior	0	0	0
Average high	0	0	0
Average	0	0	0
Average low	2	0	1
Borderline	5	0	1
Very low	2	4	2
ENI			
Attention			
Figures cancelation	28.67 (11.19)	15 (14.47)	17.50 (13.23)
Letters	20.67 (10.71)	12.25 (11.81)	19.75 (16.60)
Backward digits	4.22 (1.39)	4.25 (1.26)	1.50 (1.91)
Memory			
Auditory list, coding	28.44 (7.30)	17.50 (9.04)	21.75 (8.14)
Auditory list, recall	6.44 (3.13)	4 (2.45)	6.25 (2.06)
Complex figure, recall	8.33 (2.87)	4.50 (3.70)	4.50 (3.87)
Visuospatial			
Complex figure, copy	11.33 (3.84)	6.25 (5.19)	8.50 (4.73)
Language			
Naming	11.56 (3.05)	10 (1.83)	8.25 (4.27)
Instructions	8.22 (0.97)	6.25 (1.71)	7.63 (1.25)
Word-picture matching	14.56 (0.73)	13.13 (2.78)	11.50 (5.74)

Values are presented as mean (standard deviation), except for IQ Classification whose values are presented as frequencies.

### 3.6 Correlations and associations with language lateralization

Figure 5 illustrates the correlations among numerical variables. The LI showed significant negative correlations with the WISC-IV Working Memory Index (*ρ* = −0.60, 95% CI [−0.84, −0.17], *p* = 0.01), the ENI figures cancelation test (*ρ* = −0.49, 95% CI [−0.78, −0.01], *p* = 0.05), and the complex figure recall test (*ρ* = −0.53, 95% CI [−0.80, −0.06], *p* = 0.03). However, after adjustment for multiple comparisons using Holm’s method, these associations did not remain statistically significant. A complete correlation matrix with corresponding *p*-values is provided in Supplementary Table S3.

**Figure 5:**
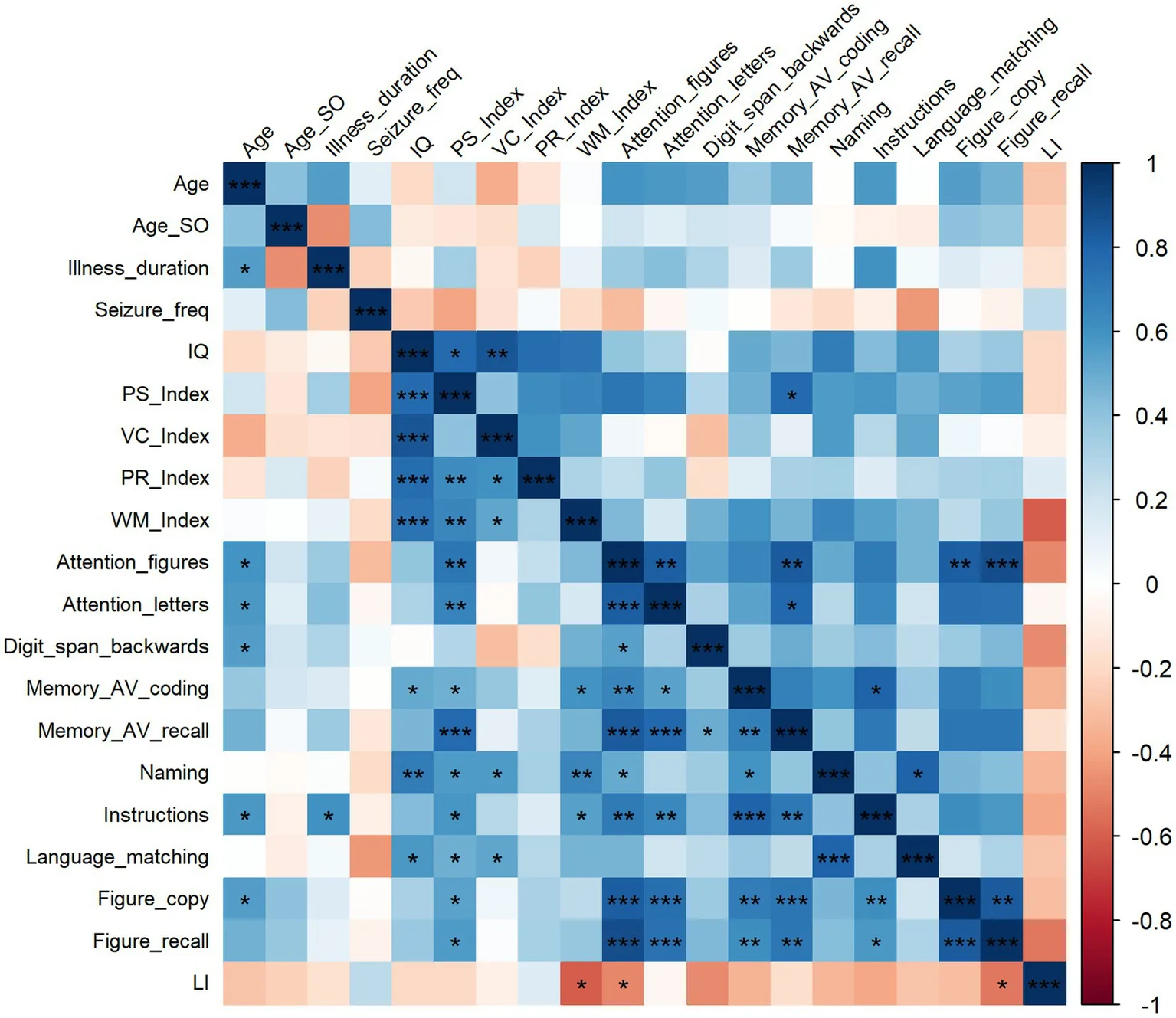
Correlation matrix of the study variables. Darker blue squares indicate stronger positive Spearman’s rho values, and darker red squares indicate stronger negative values. Significant correlations are marked by stars (*p* < 0.001 ***, *p* < 0.01 **, *p* < 0.05 *) and those above the diagonal are adjusted for multiple comparisons using Holm’s method. Age_SO, age of seizures onset; Seizure_freq, seizure frequency; PS_Index, processing speed index, VC_Index, verbal comprehension index, PR_Index, perceptual reasoning index, WM_Index, working memory index, Memory_AV, auditory list; LI, laterality index.

Table 5 summarizes the associations between language organization typicality and categorical variables. No statistically significant associations were observed. IQ classification was the variable that showed the association that was closest to significance (*p* = 0.14).

**Table 5:** Language lateralization typicality association with other categorical variables.

Variable	*p*
Sex	0.43
Education	0.37
Handedness	0.38
Seizure type	0.41
Number of antiseizure medications	0.67
Type of lesion	0.34
Hemisphere of epileptogenic zone	1.00
IQ classification	0.14

Fisher’s exact test was performed for all cases, given the sample size and the fact that none of the contingency tables met the assumptions for a Pearson’s chi-squared test.

### 3.7 Regression models

After correction for multiple comparisons, neither variable remained significantly correlated with LI. Given the limited sample size, reduced statistical power may have contributed to the attenuation of these associations. Therefore, the three variables that showed significant correlations with LI prior to adjustment were entered into separate simple linear regression models (Figure 6).

**Figure 6:**
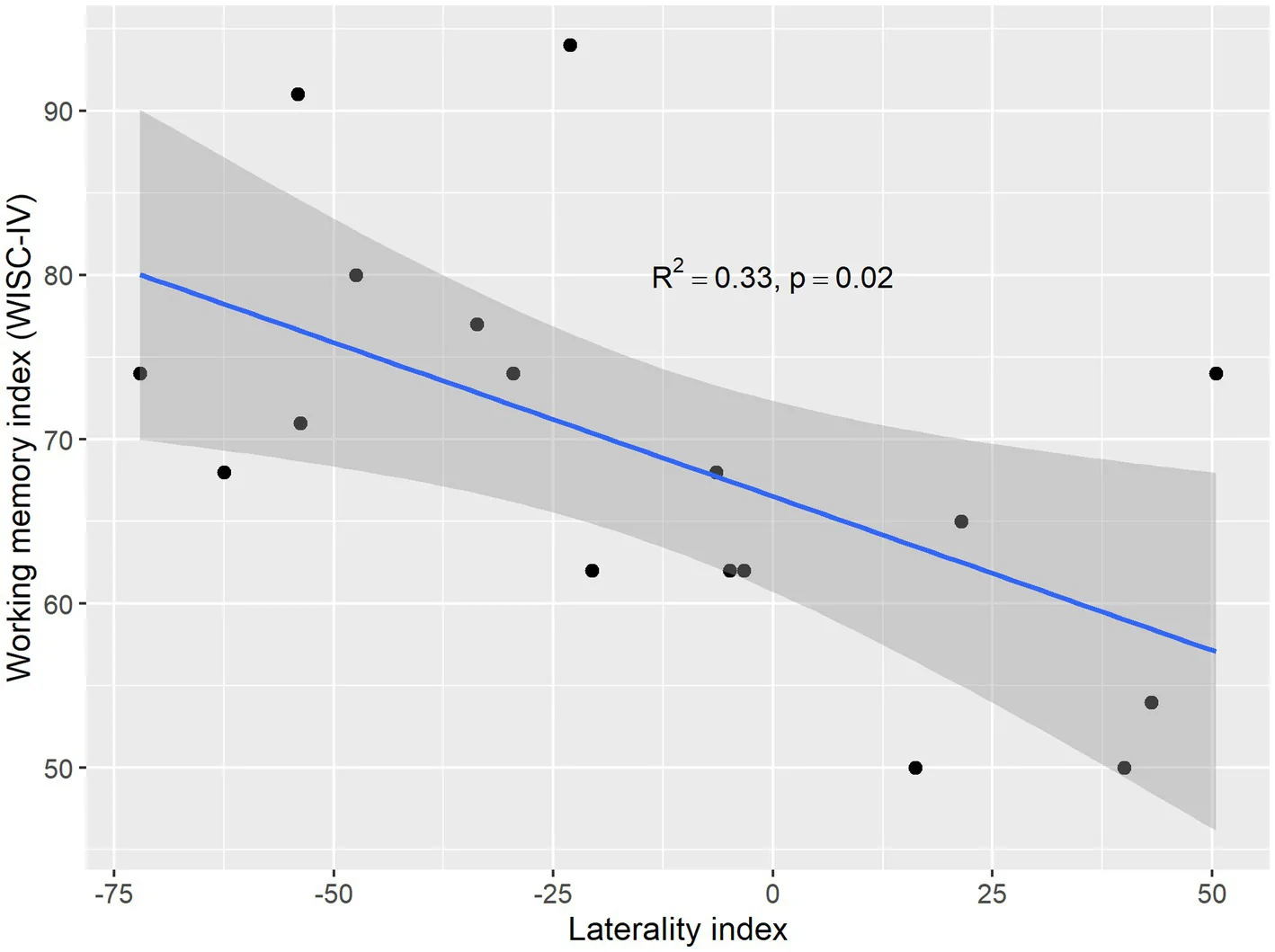
Simple linear regression model introducing the Working Memory Index from the WISC-IV as the outcome variable.

In the model predicting the WISC-IV Working Memory Index, LI accounted for 33% of the variance (*R*^2^ = 0.33, *β* = −0.19, *p* = 0.02). Conversely, neither the model predicting ENI figures test performance (*R*^2^ = 0.18, *β* = −0.15, *p* = 0.09) nor the one predicting ENI complex figure recall performance (*R*^2^ = 0.22, *β* = −0.04, *p* = 0.06) reached significance.

## 4 Discussion

The current investigation aimed to determine the correlation between various cognitive domains and the atypical cortical organization of language observed in individuals diagnosed with DRFE. This cross-sectional study included pediatric patients with DRFE who were undergoing Phase I of the presurgical evaluation for epilepsy surgery, consisting of non-invasive diagnostic studies including fMRI with a language paradigm and neuropsychological assessment. Therefore, the findings and characteristics of this population should be interpreted within the specific context of this stage of the presurgical evaluation and not according to their subsequent progression to later phases, the performance of invasive procedures, or a specific surgical outcome.

As this study focused on pediatric patients, it has implications for understanding language neurodevelopment and other cognitive functions. A higher proportion of patients’ atypical language organization in the right hemisphere or bilaterally was noted compared to typical literature reports. Additionally, patients with a bilateral atypical organization showed more pronounced cognitive deficits. It is important to note that our sample had, as a particular feature, a globally reduced cognitive profile, with impairments observed across all participants. This represents a relevant particularity of the sample and should not be interpreted as representative of the cognitive profile of the broader pediatric population with DRFE, nor necessarily of all pediatric patients evaluated as candidates for epilepsy surgery. Rather, we consider that the specific clinical and contextual characteristics of our population may have jointly or synergistically contributed to the magnitude of the cognitive impairment observed. These potential factors, including disease burden and duration, and the specific characteristics of access to presurgical evaluation and surgical treatment in our regional context, will be discussed below.

Furthermore, correlational analyses suggested a potential association between ALL and tasks involving working memory, potentially explaining part of the variance in such tasks; however, these findings did not remain significant after correction for multiple comparisons and should be interpreted with caution. These findings will be discussed within the framework of existing evidence and theoretical models, considering their clinical significance and acknowledging study limitations. Suggestions for future research directions in investigating language atypicality in pediatric and adult patients with DRFE will also be provided.

### 4.1 Atypical lateralization

The typical language organization pattern, characterized by left hemisphere dominance, is predominant in adults and individuals without neurological disorders (40, 41). 90 to 95% of healthy individuals are estimated to have this pattern, primarily involving the left inferior frontal gyrus and temporoparietal junction (1–3). In our investigation, almost 80% of patients exhibited ALL, either bilateral or with right hemisphere dominance. This finding aligns with prior studies that have noted a notable increase in ALL among pediatric individuals with DRFE (32). Although fMRI results may occasionally differ from invasive techniques (42), our study corroborated some findings. Specifically, three patients underwent the Wada test, two underwent cortical mapping with stereo encephalography (SEEG), and three underwent surgery without experiencing postoperative language impairment.

Among the patients with ALL, 4 out of 13 displayed almost symmetrical activation of both hemispheres in response to audio verbal stimuli. While several studies propose a structural predisposition for language lateralization, which may emerge even before birth (43–46), the organization pattern appears inconsistent in the pediatric population. The relationship between language function and cortical activity may be more complex during early neurodevelopment, suggesting increased involvement of both brain hemispheres. Indeed, it has been suggested that a phenomenon known as equipotentiality exists during early life stages, wherein both hemispheres could contribute to linguistic processing (47). The pattern of bilateral language organization during childhood, followed by its gradual decline in favor of hemispheric specialization, has been observed in fMRI studies. This process is typically fully established by approximately age 10 (48–50), although it may sometimes extend into early adulthood (9). This observation aligns with and partially supports our finding regarding bilateral language distribution.

Although the neurotypical model of the gradual establishment of language asymmetry may partially account for the increasing number of individuals with bilateral language organization, it is insufficient to fully explain our findings. Fifty-three percent of the patients exhibited predominant right-hemisphere activation. We propose that the increase in right-sided language lateralization may be related to DRFE. Furthermore, we suggest that this phenomenon is associated with left-hemisphere involvement, as the epileptogenic zone (EZ) was confined to the left hemisphere in 15 out of 17 patients, compared to only 2 cases with right-sided involvement. Importantly, we argue that this distribution was not the result of convenience sampling but rather reflects the natural composition of the sample influenced by uncontrolled clinical variables. This interpretation is further supported by evidence showing that atypical language lateralization becomes increasingly likely in the presence of cumulative clinical risk factors: in a mega-analysis of 914 individuals with focal epilepsy, 80% of patients with four or more predictors, such as left-hemisphere epilepsy, non-right-handedness, longer epilepsy duration, and early acquired left-hemisphere injury, showed atypical lateralization, compared with only 19% of those with none (51). Given that our cohort combines several of these risk factors (predominantly left-sided epileptogenic zones, long-standing disease, and frequent structural lesions), the elevated prevalence observed here likely reflects this cumulative clinical burden rather than a departure from expected patterns.

Developmental neuroplasticity provides an important framework for interpreting the high prevalence of atypical language organization in this cohort, but atypical lateralization should not be considered synonymous with successful functional compensation. Previous research has suggested that early brain injury, particularly in the left hemisphere, may lead to a compensatory shift toward the contralateral hemisphere for language functions, a phenomenon that has been observed across various pathological conditions, including epilepsy (32, 48, 52, 53). A recent systematic review and meta-analysis found that earlier onset of focal epilepsy is associated with a small but significant shift toward right hemisphere language representation, with a nonlinear developmental pattern consistent with greater plastic potential during early childhood; nevertheless, age at seizure onset alone had limited predictive value for an individual patient’s language organization (54). Similarly, in children with recent onset epilepsy, the relationship between epilepsy duration and language dominance depends on age at onset, indicating that disease related reorganization interacts with the maturational state of the language system (55). Of note, in our study most cases (14 out of 17) presented macroscopic brain parenchymal damage, all associated with pre- or perinatal developmental alterations. Although the sample size limits the strength of our inferences, we found no evidence suggesting that atypical lateralization was associated with age at seizure onset. In fact, patients with typical language organization exhibited an earlier onset of seizures.

Functional connectivity studies further suggest that age and epileptogenic focus location influence how language networks redistribute processing across ipsilateral and contralateral regions (56). The language phenotype observed in pediatric drug-resistant focal epilepsy likely reflects the interaction of developmental stage, timing and location of the underlying cerebral insult, epileptogenic zone laterality, seizure burden, and duration of epilepsy. Importantly, right sided or bilateral recruitment may represent effective adaptive reorganization in some patients, but in others it may reflect persistence of a less specialized developmental organization or inefficient network reconfiguration. Recent pediatric evidence showing that language network reorganization can coexist with compromised cognitive functioning reinforces the need to distinguish neural reorganization from successful behavioral compensation (57).

### 4.2 Cognitive deficits

Although the pathophysiological mechanisms underlying DRFE may negatively impact intellectual development and emerging cortical functions, not all reported cases exhibit impairments as severe as those observed in the present sample. Therefore, our findings should not be considered representative of the disease in its early stages, but rather reflective of a specific clinical context (58). We consider that the cognitive profile of our sample is influenced by symptom severity and by the impact of seizures on patients’ functional abilities and activities of daily living, as well as on their caregivers. In this context, the magnitude of the clinical and functional burden may constitute one of the main characteristics of this population, particularly in patients whose clinical condition warrants consideration of epilepsy surgery as a priority therapeutic option. Besides, some other features of our sample must be considered, such as that most participants (14 out of 17) displayed macroscopic alterations of brain tissue associated with pre or perinatal development disturbance, while only four patients lacked an identifiable structural lesion. Likewise, age of seizure onset, epilepsy duration and yearly ictal frequency might jointly contribute to the accumulated burden of the disease and, potentially, to the observed cognitive impairment.

This is particularly relevant considering that the estimated time between diagnosis and surgical intervention for DRFE in Latin America can exceed 20 years (59). A longer interval between the diagnosis of drug-resistance and access to presurgical assessment or surgery itself might contribute to a greater accumulation of clinical and functional burden before patients reach these stages of care. Taken together, these factors could help explain the particular cognitive profile observed in our sample. However, given the cross-sectional design and the limited sample size, these variables should be regarded as potential contributing factors rather than causal determinants of cognitive performance.

Consistent with previous literature (13, 21, 22), our sample of pediatric DRFE patients showed considerable cognitive deficits in practically all domains. While not all pediatric DRE patients underperform on cognitive tests relative to healthy children, previous research has shown that the earlier the age of seizures onset, the more likely cognitive impairment is to be present (13, 21, 22); however, we found no correlation between age of seizure onset and any of the cognitive measures after multiple comparisons adjust in our study. Although the small sample size is an important limitation across the whole study, one possibility is that the distribution of ages of seizure onset was skewed toward earlier ages. Indeed, only three patients had a seizure onset older than 6 years old, which could have masked the effect of this variable on cognitive performance. This particular age of seizures’ onset distribution should be considered when interpreting the absence of significant associations in our sample, and it should be interpreted together with the duration and accumulated burden of the epilepsy, the frequency of seizures and the structural features of the disease, factors that might interact during neurodevelopmental critical periods. Therefore, we consider that the impaired cognitive performance present in our sample may reflect the interaction of multiple clinical and contextual characteristics, rather than the isolated effect of a unique variable.

One of those clinical features that should be considered when interpreting cognitive performance is the burden of antiseizure medication. All participants in the present study were receiving antiseizure medication polytherapy, with 70.58% taking three or four concurrent medications, which represents a potentially relevant source of cognitive variability. Given the relevance of this factor, in an exploratory *post hoc* analysis, we descriptively examined cognitive scores according to the number of concurrent antiseizure medications. As was the case with LI, we did not perform inferential statistics to compare the groups since their size were small. Still, scores seemed to be higher across all assessed domains in patients receiving treatment regimens of two antiseizure medications than in patients receiving treatment regimens of three or four antiseizure medications (see scores in Supplementary Table S4). This pattern should be regarded as hypothesis-generating and does not establish an independent effect of medication burden.

The number of antiseizure medications may be implicated in the cognitive profiles observed as, indeed, higher antiseizure medication burden has been associated particularly with poorer attention, executive functioning, processing speed and working memory (60–62), although the magnitude of these effects varies considerably according to the specific agent, dose, titration schedule, individual cognitive vulnerability and other clinical factors (63–65). Particularly, antiseizure drugs like topiramate (66, 67), zonisamide (68) and carbamazepine (67) are among the drugs with more recognized negative effects on cognition. However, topiramate and carbamazepine were not very commonly used in our sample (2 and 1 participants, respectively), and zonisamide was not used at all. As there was an absence of monotherapy, and small and heterogenous exposure groups, it was not possible to say if specific drugs had deleterious effects on cognition. Moreover, information concerning dose, serum concentration, treatment duration, and pharmacodynamic interactions was not available. Medication-related effects therefore cannot be separated conclusively from the effects of epilepsy severity, cortical organization, or other clinical characteristics that may have contributed to the observed findings.

One interesting observation is the relationship between language lateralization and cognitive performance. Patients with a more bilateral organization of language seemingly had a worse cognitive performance than those with a more lateralized organization, whether left- or right-sided. In other words, there seems to be a U-shaped relationship between language lateralization and cognitive performance in most measures (excluding tasks for working memory and language), where test scores tend to decrease together with hemispheric dominance for language. This result resembles a previous report on healthy adults where weaker hemispheric lateralization (i.e., more bilateral language activity) was associated with poorer scores on memory, language, and visuospatial cognitive tasks when compared to lateralized individuals (69). While that study was performed with young adults, this is still relevant due to the developmentally sensible nature of language lateralization.

There seems to be an initial less lateralized (or more bilateral) language activity during early childhood, which tends to lateralize to the left hemisphere with age, peaking around 20–25 years old. After that peak, lateralization begins to decrease (9). Additionally, better performance on language tasks is correlated to a more lateralized language activity (70–72). As cognitive skills get refined during development, this is accompanied by a strengthening in hemispheric dominance (1). As such, one could expect an age-dependent relationship between the degree of lateralization and cognitive performance, suggesting that a lasting bilateral language organization during the first two decades of life would be linked to poorer performance on cognitive tasks. A disruption of the lateralization process during childhood and adolescence could delay the shift from bilateral organization to lateralized organization (whether typical or atypical).

Regardless of language lateralization changes across development, conflictive evidence suggests positive and negative relationships between cognitive performance and ALL. For example, Löring et al. (73) found that left mesial temporal epilepsy adult patients with a right dominance for language had lower scores in visuospatial tasks than patients with a typical left-dominant hemisphere. At the same time, those with bilateral activity also performed worse in these tasks than patients with left laterality, but only when they had a non-dextral handedness. Noteworthy, the authors reported that while patients with earlier age of onset did have a lower score across several measures, they did not find a significant effect, and so it was not clear that the age of seizure onset mediated the laterality-cognition relationship.

Conversely, Everts (31) found that ALL (bilateral and right-sided) in pediatric FE was correlated with better verbal memory performance. While ALL might benefit some cognitive functions and be linked with worse performance on others, a possible alternative explanation for this lack of convergence in previous evidence could be that lateralized activity has been operationalized as a static measure. Recent research (74) has shown that brain networks supporting different cognitive functions display dynamic lateralization, with both intra- and inter-hemispheric fluctuations and reversals. This nuances all the results from studies into the brain lateralization of cognitive functions, both in physiological and pathological states, and poses the need to address the temporal dynamics of atypical language lateralization in epilepsy. Further research is needed to understand better the effects on cognition of both the lack of lateralization and the presence of atypical lateralization of language networks, especially when mediated by pathological processes that disrupt typical development, such as in pediatric DRFE.

### 4.3 Working memory-ALL exploratory relationship

Although initial analyses suggested associations between language lateralization and performance in working memory, attention, and visuospatial memory, these differences did not remain significant after correction for multiple comparisons, limiting the robustness of these findings. Further, in the regression models, language lateralization showed a significant effect only on working memory, accounting for 33% of its variance. In this context, findings regarding working memory are discussed below in relation to potential functional reorganization mechanisms associated with atypical language lateralization but also stressing the limitations of the present study and the exploratory nature of the analyses.

When describing the cognitive performance in our sample, patients with atypical right lateralization seemed to have higher scores in audio verbal working memory tasks. While this finding could suggest potential plasticity mechanisms enabling cortical reorganization and functional preservation, some other explanations remain possible, such as influence from dissimilar lesion features, developmental stage, seizure burden, global cognitive and premorbid functioning, and measurement variability. Additionally, it is not possible to ascertain whether RAL contributed to the performance in working memory tasks, whether premorbid working memory differences influenced the lateralization patterns, or whether they were both shaped by some others clinical and/or developmental factors due to the cross-sectional design used.

Still, an association between ALL and working memory performance might be biologically plausible due to shared or overlapping networks. The multi-component model of working memory proposed by Baddeley (75, 76), which includes the phonological loop, visuospatial sketchpad (temporary storage), episodic buffer (temporary storage of multimodal information and short- and long-term memory binding), and the central executive (manipulation and organization), has been widely accepted and supported by empirical evidence. Brain activation patterns for these components have been observed to vary across different age groups, particularly in individuals experiencing prolonged maturation processes during childhood (77). There exists a possibility then that the age of the patients may facilitate plasticity processes in general, including migration toward the opposite hemisphere or expansion of activation areas. Specifically, the sensorimotor circuit, the inferior parietal lobule, and the inferior frontal gyrus of the left hemisphere have been linked to the functioning of the phonological loop (78). Conversely, the visuospatial sketchpad, considered the visual counterpart of the phonological loop, has been associated with activation in the right hemisphere (77). This interhemispheric functional asymmetry (79) indicates that the verbal aspects of working memory are primarily associated with the prefrontal cortex of the left hemisphere, while visuospatial aspects are linked to the prefrontal regions of the right hemisphere, although these areas are probably just prominent hubs within more distributed frontoparietal networks (80–82).

In children with predominantly left-hemisphere pathology, cortical plasticity may involve intrahemispheric redistribution, bilateral recruitment, or a shift of language-related activity toward the contralateral hemisphere. It is likely that if there are mechanisms at play facilitating the preservation of language functions, they also extend to areas involved in audio verbal aspects of working memory. Although audio verbal working memory is typically regarded as an emergent function of prefrontal activity, it also relies on the activation of other regions associated with language processing. These include the temporoparietal junction (Wernicke’s area) for the maintenance of sounds and words in memory and frontal areas such as the inferior frontal gyrus for the repetition of verbal information we intend to remember (77–79, 83, 84). Because language and verbal working memory share neural resources, a possibility is that their functional organization may change in parallel.

It has been suggested that verbal working memory, primarily activated in the left hemisphere, undergoes reorganization more readily than visuospatial working memory (85). This predominance of reorganization in left-sided lesions is believed to be linked to the processing of verbal versus visuospatial information, with the latter maturing and consolidating earlier during sensitive stages of cognitive development (86). In contrast, linguistic development typically begins with concrete concepts and progresses toward more abstract ones, rendering it more prone to reorganization due to its dynamic and continuous nature extending into early adulthood. Furthermore, working memory has been proposed to predict high-complexity processes such as language (87).

From a network perspective, atypical language lateralization should not be interpreted simply as a transfer of function from one hemisphere to the other. Pediatric epilepsy studies demonstrate that altered language organization may involve reduced intrahemispheric connectivity within the left hemisphere and reduced interhemispheric connectivity together with increased recruitment of right hemisphere networks; importantly, these network properties are also associated with cognitive performance (88, 89). Evidence in focal epilepsy further indicates that language and working memory dysfunction can arise from disruption of large-scale network integration rather than from dysfunction of a single cortical region (90). The relationship observed in our cohort between language lateralization and working memory performance may reflect a shared process of network reorganization or compensatory redistribution rather than a direct causal effect of hemispheric dominance on working memory. A hypothetical conceptual model describing the relationships that may underlie the cognitive and lateralization patterns observed is shown in Figure 7.

**Figure 7:**
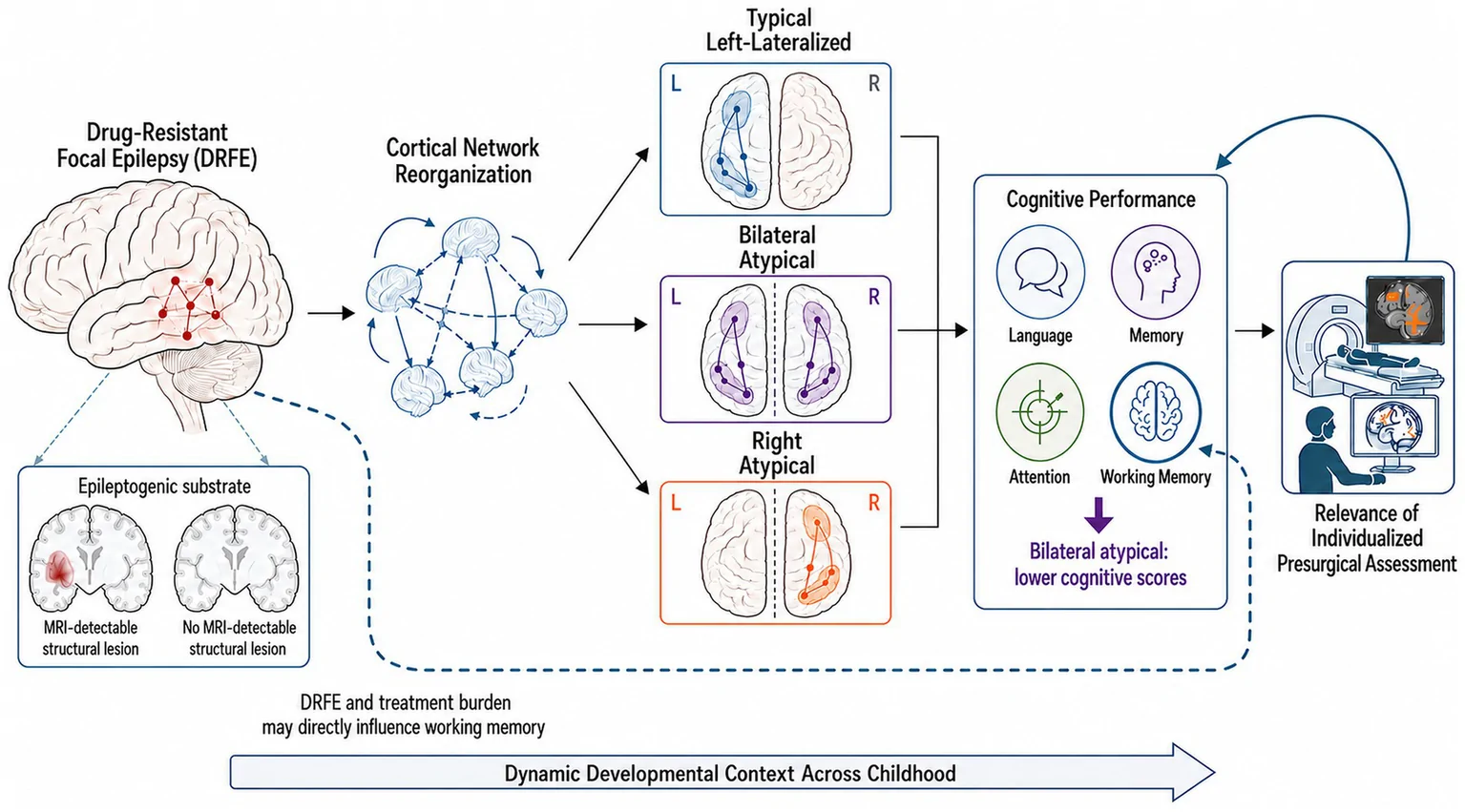
Hypothetical conceptual model linking epileptic pathology, cortical reorganization, language lateralization, and cognitive performance in pediatric drug-resistant focal epilepsy (DRFE). The epileptogenic substrate may include an MRI-detectable structural lesion or have no identifiable structural correlate on conventional MRI. Epilepsy-related neuroplastic reorganization may contribute to typical left-lateralized, bilateral atypical, or right-atypical language configurations, which may, in turn, be associated with cognitive performance. In the present sample, the bilateral atypical group showed descriptively lower scores across several cognitive domains; given the small group sizes and absence of inferential between-group comparisons, this pattern should not be interpreted as a statistically established or causal effect of bilateral lateralization. Similarly, the observed associations between rightward lateralization and working memory should be considered exploratory because they did not consistently remain significant after correction for multiple comparisons. The heavier dashed pathway indicates that DRFE and treatment burden, including antiseizure medication polytherapy, may also contribute to working-memory performance independently of language lateralization. These relationships occur within a dynamic developmental context across childhood. Given the relevance these links may have in the context of epilepsy surgery and its outcomes, an individualized presurgical assessment addressing both cognitive performance and the language lateralization pattern is of the utmost importance to uncover and characterize these complex relationships, contextualizing them within each individual clinical scenario. All arrows represent hypothesized or clinically informed relationships rather than demonstrated causal pathways.

Still, given that the correlations did not remain significant after correction for multiple comparisons, this network level interpretation should be considered hypothesis-generating. Besides, the present study did not include working memory fMRI paradigms, functional connectivity analyses, or longitudinal measurements. Consequently, our findings cannot demonstrate that working memory functions reorganized alongside language networks or that atypical lateralization preserved working memory performance. Therefore, ALL should not be interpreted from these findings as inherently protective or deleterious. Rather, the observed pattern identifies a candidate association for future hypothesis-driven research. Larger longitudinal studies should examine this relationship using prespecified analyses, appropriate adjustment for multiple comparisons, specific working memory fMRI paradigms, ideally functional connectivity measures, and control for relevant clinical and developmental variables. Such studies could determine whether language and working memory networks undergo coordinated reorganization and whether their relationship has meaningful implications for presurgical assessment and postoperative cognitive outcomes.

In this context, it must be underscored that resective surgery is emerging as the most effective treatment option for DRFE to reduce the impact on patients’ lives and society (91). This is consistent with current evidence indicating that epilepsy surgery is the treatment of choice for drug-resistant epilepsy and offers the highest likelihood of sustained seizure freedom in appropriately selected patients, with benefits that extend to quality of life and behavioral outcomes in pediatric populations (92). This is especially critical in low- and middle-income countries, where the time between diagnosis and access to surgical intervention is estimated to be, on average, 20 years for adults and 10 years for pediatric populations (59). In Latin America, these delays appear to be driven by the geographic centralization of epilepsy surgery programs, limited diagnostic infrastructure, under-referral, economic barriers, shortage of specialists, and persistent misconceptions about surgical risks among patients and providers (93). Delayed referral is clinically relevant because longer epilepsy duration is associated with worse surgical outcomes, lower quality of life, and greater morbidity and mortality, reinforcing current recommendations to refer patients for surgical evaluation as soon as drug resistance is established (94). These challenges also highlight the fact that our final sample size, although small, is not negligible since delay in recognizing drug resistance and reaching surgical candidacy substantially reduce the number of pediatric patients who arrive at comprehensive epilepsy programs and, consequently, the number available for research (95).

### 4.4 Clinical implications for presurgical assessment

The high frequency of atypical language lateralization observed in this sample reinforces the importance of characterizing language organization individually during the presurgical evaluation of pediatric patients with drug-resistant focal epilepsy. Clinical variables such as handedness, lesion location, and age at seizure onset may inform expectations regarding language dominance but cannot substitute for direct functional assessment (9). This consideration may be particularly relevant when the epileptogenic zone involves the left hemisphere, as occurred in most patients in the present sample, because cortical reorganization may shift or extend language-related activity toward contralateral or bilateral regions (96).

Presurgical language fMRI may contribute to this assessment by identifying task-related activation and estimating the relative involvement of both hemispheres across receptive, expressive, and mixed language paradigms. Nevertheless, the laterality index should be interpreted as one component of a multimodal evaluation rather than as an isolated indicator of surgical risk (97). fMRI atypical or bilateral activation can reflect functional reorganization, but it may also be influenced by developmental stage, task selection, performance during scanning, motion, analysis thresholds, or diffuse network recruitment (26). Moreover, fMRI demonstrates task-related activation but does not directly establish whether an activated region is indispensable for language performance. When fMRI findings are inconclusive, discordant with other clinical information, or located close to the proposed resection, complementary procedures such as the Wada test (98), stereo-electroencephalography (SEEG) stimulation (99) or intraoperative cortical mapping (100, 101) may therefore remain necessary, depending on the patient’s age, clinical characteristics, and planned surgical approach.

Comprehensive neuropsychological assessment provides essential information for interpreting the functional relevance of the neuroimaging findings. Presurgical evaluation should establish a baseline profile that includes receptive and expressive language, verbal and visual memory, attention, executive functions, visuospatial abilities and intellectual level (25). Language lateralization should not be considered a proxy for the integrity or efficiency of these functions. For example, the descriptively lower performance observed among patients with bilateral language organization suggests that a more distributed activation pattern does not necessarily indicate greater cognitive reserve. Similarly, the exploratory association between rightward lateralization and higher working-memory scores should not be interpreted as evidence that atypical lateralization is inherently protective. Instead, laterality findings should be examined in relation to the patient’s broader cognitive profile and the anatomical and electrophysiological characteristics of the epileptogenic network.

From the perspective of surgical planning, atypical language organization may alter the distribution of functional risk (102). Rightward reorganization could transfer part of that risk to the right hemisphere, whereas bilateral representation may indicate that relevant language processes are supported by distributed regions in both hemispheres. Consequently, identifying only the dominant hemisphere may be insufficient when the proposed resection approaches task-responsive language cortex. The spatial relationship between the epileptogenic zone, the planned resection, and frontal and temporoparietal language regions should be evaluated for each patient. When clinically available, information regarding functional connectivity (103) and the integrity of language-related white-matter pathways (104, 105) may further contribute to this individualized network-level assessment.

The integration of fMRI and neuropsychological findings may also improve presurgical counseling. Establishing the patient’s cognitive strengths and vulnerabilities can support a more individualized discussion of potential postoperative changes and help identify functions that require closer monitoring (106, 107). The baseline evaluation can subsequently serve as a reference for postoperative follow-up and for planning cognitive, educational, or language rehabilitation when necessary. This approach is particularly important in children and adolescents, because surgical outcomes must be considered within the context of continuing neurodevelopment rather than exclusively in terms of immediate postoperative deficits.

These clinical implications should nevertheless be interpreted within the limitations of the present study. The cross-sectional design, small sample size, heterogeneous etiologies, and absence of systematic postoperative follow-up prevent us from determining whether the observed lateralization patterns predict surgical outcomes. Therefore, the present findings should not be used to define surgical candidacy, establish resection boundaries, or infer cognitive prognosis from laterality categories alone. Rather, they support the inclusion of individualized language mapping and comprehensive neuropsychological assessment within a multidisciplinary presurgical evaluation. Future longitudinal studies should determine whether the joint consideration of language lateralization, cognitive performance, structural abnormalities, and electrophysiological findings improves the prediction of postoperative language and cognitive outcomes.

### 4.5 Future directions

As language lateralization is an extended ongoing process during childhood and possibly adolescence, it would be essential to assess whether the language lateralization distribution observed in our sample would persist during adulthood, as it could be possible that the rates of ALL changes across age. Adults were intentionally not included because this study was designed to examine the relationship between language organization and cognition during active neurodevelopment rather than across the lifespan. Language lateralization and the functional architecture supporting language continue to mature throughout childhood and adolescence, and, in focal epilepsy, the influence of epilepsy on language dominance is itself developmentally dependent. Recent evidence indicates that earlier seizure onset is associated with a modest rightward shift in language lateralization at the group level, while the effect of epilepsy duration varies according to the developmental stage at which seizures begin (54, 55). Therefore, adults with childhood onset epilepsy cannot be considered developmentally equivalent comparators for children: despite sharing an early disease onset, they have undergone subsequent maturation of language networks together with substantially longer cumulative exposure to seizures, antiseizure treatment, and epilepsy related network reorganization. Moreover, the neuropsychological protocol used in this study (WISC IV and ENI) was specifically selected for pediatric assessment; inclusion of adults would require different instruments and normative frameworks, limiting direct comparability of cognitive measures. An adult cohort would address a complementary lifespan question but would not constitute an appropriate internal comparison group for the present pediatric presurgical cohort.

Further, the relationship between ALL and cognition could also be explored through its biological and network basis using functional connectivity measures. The temporal dynamics of language lateralization in pediatric and adult DRFE should be explored to uncover its nuances and possibly more comprehensive relationship with other cognitive domains. Also, post-surgical follow-up neuropsychological assessment should be included in future analyses to better understand the links between a postulated dynamic reorganization process in the language cortical representation and cognitive performance. Finally, convergent measures could be obtained from measuring indexes of white matter integrity and its relationship to both language lateralization and cognitive test scores.

### 4.6 Limitations

The sample size should be expanded to achieve more robust generalizations and be able to detect smaller effects; however, the sample’s characteristics (pediatric DRFE patients, candidates for epilepsy surgery) make it particularly challenging to obtain large samples. Additionally, it was decided not to perform a region of interest (ROI) analysis due to time constraints, which could have increased the precision of language lateralization measurements. Another aspect to consider is the non-inclusion of a healthy control group, which was limited by the study budget. A healthy control group would allow for a more certain establishment of which effects specifically correspond to DRFE and how language lateralization varies in physiological and pathological states in this population without a mature nervous system. On the other hand, it would be important in future studies to establish a convergence between fMRI results and the Wada test to confirm the laterality data, which can be challenging given the invasive nature of the test that is sometimes preferred to be avoided in very young patients.

In clinical settings, fMRI is performed for localization and presurgical planning, and therefore no behavioral data is tracked during this procedure; access to behavioral data could potentiate and validate the fMRI data. There are also some technical aspects that are intrinsic to language lateralization mapping with fMRI, such as the somewhat arbitrary cutoffs that define what is and what is not atypical. For example, a LI of 0.20 is classified as typical but an index of 0.19 would be atypical. Finally, the heterogeneity of etiological processes behind refractory epilepsy possibly influences in different ways both functional activity and cognitive outcomes; isolating and estimating the effect of such heterogeneity is a challenge that remains to be addressed.

## 5 Conclusion

For this study among pediatric patients with DRFE, almost 80% had ALL, with 53% of those ALL patients showing right lateralization and 24% exhibiting bilateral organization, with an apparent lower cognitive performance in virtually all domains for the latter group; however, this last observation needs to be confirmed through inferential statistics. Additionally, an association was found between ALL and better working memory performance. Future efforts to describe the association between ALL and cognition in a developing population, such as pediatric DRFE, require sample sizes that provide higher statistical power to elucidate potential plasticity mechanisms in response to injury and their interaction with cognitive development. These findings support integrating patient-specific language fMRI with comprehensive neuropsychological assessment during presurgical evaluation. Language laterality categories should not be interpreted as isolated indicators of functional preservation or surgical risk, but rather in conjunction with the patient’s cognitive profile, lesion characteristics, epileptogenic-zone location, and other functional-mapping findings.

## Data Availability

The original contributions presented in the study are included in the article/supplementary material, further inquiries can be directed to the corresponding author/s.
