# Supplementary material for: Relationship between atypical language lateralization and cognition in pediatric patients with drug-resistant focal epilepsy: role of working memory
Source: Front Neurol. 2026 Sep 24;17:1893275. doi: 10.3389/fneur.2026.1893275 (PMC13645585; doi:10.3389/fneur.2026.1893275)
Supplement: Supplementary file 1 [file Table_1.DOCX]

| **Table S2**  ENI tests percentile scores | | | | | | | | | | | |
| --- | --- | --- | --- | --- | --- | --- | --- | --- | --- | --- | --- |
| ID | Attention_figures | Attention_letters | Digit_span_backwards | Memory_AV_coding | Memory_AV_recall | Naming | Instructions | Language_matching | Figure_copy | Figure_recall | Typicality |
| EP01 | 2 | 5 | 63 | 1 | 2 | 50 | 9 | 37 | 16 | 16 | Bilateral atypical |
| EP02 | 0.1 | 0.1 | 37 | 5 | 0.1 | 0.1 | 50 | 2 | 0.1 | 0.4 | Right atypical |
| EP03 | 5 | 2 | 26 | 9 | 5 | 26 | 2 | 37 | 50 | 37 | Bilateral atypical |
| EP04 | 0.1 | 0.1 | 2 | 9 | 2 | 5 | 2 | 9 | 2 | 2 | Typical |
| EP05 | 0.1 | 0.1 | 16 | 5 | 5 | 50 | 16 | 75 | 0.4 | 9 | Right atypical |
| EP06 | 37 | 0.1 | 63 | 2 | 9 | 37 | 2 | 50 | 63 | 37 | Right atypical |
| EP07 | 2 | 0.1 | 5 | 9 | 0.1 | 63 | 16 | 50 | 9 | 26 | Right atypical |
| EP08 | 75 | 26 | 37 | 26 | 37 | 75 | 63 | 50 | 50 | 75 | Right atypical |
| EP09 | 50 | 9 | 50 | 91 | 63 | 84 | 63 | 50 | 75 | 63 | Right atypical |
| EP10 | 26 | 5 | 63 | 0.1 | 26 | 16 | 50 | 75 | 63 | 5 | Right atypical |
| EP11 | 16 | 50 | 9 | 37 | 63 | 75 | 63 | 50 | 26 | 63 | Typical |
| EP12 | 26 | 26 | 5 | 9 | 5 | 0.1 | 1 | 0.4 | 37 | 26 | Typical |
| EP13 | 1 | 0.1 | 63 | 0.1 | 0.4 | 63 | 1 | 50 | 0.1 | 0.1 | Bilateral atypical |
| EP14 | 0.1 | 0.1 | 5 | 0.1 | 0.1 | 9 | 0.1 | 0.1 | 0.1 | 0.4 | Bilateral atypical |
| EP15 | 50 | 5 | 26 | 50 | 26 | 84 | 63 | 50 | 75 | 37 | Right atypical |
| EP16 | 9 | 5 | 1 | 37 | 50 | 75 | 37 | 63 | 2 | 9 | Typical |
| EP17 | 37 | 5 | 26 | 16 | 0.1 | 91 | 9 | 50 | 26 | 26 | Right atypical |

AV, auditory list.

| **Table S3**  Correlation matrix of the study variables | | | | | | | | | | | | | | | | | | | | | |
| --- | --- | --- | --- | --- | --- | --- | --- | --- | --- | --- | --- | --- | --- | --- | --- | --- | --- | --- | --- | --- | --- |
|  |  | 1 | 2 | 3 | 4 | 5 | 6 | 7 | 8 | 9 | 10 | 11 | 12 | 13 | 14 | 15 | 16 | 17 | 18 | 19 | 20 |
| 1. Age | rho  p | 1  - | 0.41  1 | 0.55  1 | 0.12  1 | -0.19  1 | 0.19  1 | -0.37  1 | -0.15  1 | 0.03  1 | 0.59  1 | 0.58  1 | 0.55  1 | 0.39  1 | 0.48  1 | -0.01  1 | 0.57  1 | 0  1 | 0.56  1 | 0.48  1 | -0.28  1 |
| 2. Age_SO | rho  p | 0.41  0.10 | 1  - | -0.47  1 | 0.43  1 | -0.11  1 | -0.13  1 | -0.17  1 | 0.16  1 | 0.01  1 | 0.20  1 | 0.14  1 | 0.20  1 | 0.19  1 | 0.04  1 | -0.02  1 | -0.07  1 | -0.10  1 | 0.41  1 | 0.39  1 | -0.24  1 |
| 3. Duration | rho  p | **0.55**  **0.02** | -0.47  0.06 | 1  - | -0.23  1 | -0.04  1 | 0.34  1 | -0.15  1 | -0.22  1 | 0.10  1 | 0.37  1 | 0.42  1 | 0.31  1 | 0.15  1 | 0.37  1 | 0.02  1 | 0.60  1 | 0.05  1 | 0.14  1 | 0.10  1 | -0.16  1 |
| 4. Szr_freq | rho  p | 0.12  0.64 | 0.43  0.08 | -0.23  0.38 | 1  - | -0.26  1 | -0.40  1 | -0.16  1 | 0.03  1 | -0.19  1 | -0.31  1 | -0.05  1 | 0.04  1 | 0  1 | -0.12  1 | -0.18  1 | -0.08  1 | -0.43  1 | -0.01  1 | -0.07  1 | 0.26  1 |
| 5. FSIQ | rho  p | -0.19  0.45 | -0.11  0.67 | -0.04  0.88 | -0.26  0.31 | 1  - | **0.78**  **0.04** | **0.85**  **<0.01** | 0.77  0.06 | 0.73  0.15 | 0.40  1 | 0.31  1 | -0.02  1 | 0.50  1 | 0.46  0.31 | 0.69  0.35 | 0.43  1 | 0.58  1 | 0.33  1 | 0.38  1 | -0.20  1 |
| 6. PS_Index | rho  p | 0.19  0.46 | -0.13  0.61 | 0.34  0.18 | -0.40  0.11 | **0.78**  **<0.01** | 1  - | 0.41  1 | 0.62  1 | 0.66  0.68 | 0.72  0.19 | 0.67  0.58 | 0.30  1 | 0.49  1 | **0.78**  **0.04** | 0.57  1 | 0.58  1 | 0.48  1 | 0.53  1 | 0.57  1 | -0.20  1 |
| 7. VC_Index | rho  p | -0.37  0.14 | -0.17  0.51 | -0.15  0.57 | -0.16  0.54 | **0.85**  **<0.01** | 0.41  0.10 | 1  - | 0.60  1 | 0.53  1 | 0.05  1 | -0.03  1 | -0.30  1 | 0.38  1 | 0.11  1 | 0.56  1 | 0.29  1 | 0.52  1 | 0.07  1 | 0.02  1 | -0.08  1 |
| 8. PR_Index | rho  p | -0.15  0.58 | 0.16  0.53 | -0.22  0.39 | 0.03  0.90 | **0.77**  **<0.01** | **0.62**  **0.01** | **0.60**  **0.01** | 1  - | 0.31  1 | 0.25  1 | 0.40  1 | -0.17  1 | 0.14  1 | 0.32  1 | 0.34  1 | 0.15  1 | 0.29  1 | 0.32  1 | 0.34  1 | 0.14  1 |
| 9. WM_Index | rho  p | 0.03  0.92 | 0.01  0.97 | 0.10  0.71 | -0.19  0.47 | **0.73**  **<0.01** | **0.66**  **<0.01** | **0.53**  **0.03** | 0.31  0.22 | 1  - | 0.45  1 | 0.17  1 | 0.48  1 | 0.60  1 | 0.46  1 | 0.65  0.76 | 0.55  0.69 | 0.47  1 | 0.26  1 | 0.39  1 | -0.60  1 |
| 10. Attntn_fig | rho  p | **0.59**  **0.01** | 0.20  0.44 | 0.37  0.14 | -0.31  0.22 | 0.40  0.11 | **0.72**  **<0.01** | 0.05  0.84 | 0.25  0.33 | 0.45  0.07 | 1  - | **0.83**  **0.01** | 0.54  1 | 0.66  0.67 | **0.84**  **<0.01** | 0.51  1 | 0.70  0.27 | 0.46  1 | **0.83**  **0.01** | **0.89**  **<0.01** | -0.49  1 |
| 11. Attntn_letters | rho  p | **0.58**  **0.02** | 0.14  0.60 | 0.42  0.09 | -0.05  0.86 | 0.31  0.22 | **0.67**  **<0.01** | -0.03  0.91 | 0.40  0.11 | 0.17  0.50 | **0.83**  **<0.01** | 1  - | 0.33  1 | 0.53  1 | **0.79**  **0.03** | 0.29  1 | 0.64  0.92 | 0.20  1 | 0.75  0.09 | 0.75  0.10 | -0.04  1 |
| 12. DS_backwards | rho  p | **0.55**  **0.02** | 0.20  0.44 | 0.31  0.23 | 0.04  0.86 | -0.02  0.95 | 0.30  0.25 | -0.30  0.23 | -0.17  0.51 | 0.48  0.05 | **0.54**  **0.02** | 0.33  0.20 | 1  - | 0.36  1 | 0.49  1 | 0.35  1 | 0.44  1 | 0.27  1 | 0.36  1 | 0.44  1 | -0.47  1 |
| 13. AV_coding | rho  p | 0.39  0.13 | 0.19  0.48 | 0.15  0.56 | 0  0.99 | **0.50**  **0.04** | **0.49**  **0.05** | 0.38  0.13 | 0.14  0.59 | **0.60**  **0.01** | **0.66**  **<0.01** | **0.53**  **0.03** | 0.36  0.16 | 1  - | 0.69  0.40 | 0.59  1 | **0.81**  **0.02** | 0.37  1 | 0.70  0.32 | 0.61  1 | -0.35  1 |
| 14. AV_recall | rho  p | 0.48  0.05 | 0.04  0.89 | 0.37  0.15 | -0.12  0.64 | 0.46  0.06 | **0.78**  **<0.01** | 0.11  0.66 | 0.32  0.21 | 0.46  0.06 | **0.84**  **<0.01** | **0.79**  **<0.01** | **0.49**  **0.04** | **0.69**  **<0.01** | 1  - | 0.39  1 | 0.72  0.21 | 0.25  1 | 0.73  0.17 | 0.72  0.18 | -0.18  1 |
| 15. Naming | rho  p | -0.01  0.98 | -0.02  0.94 | 0.02  0.95 | -0.18  0.49 | **0.69**  **<0.01** | **0.57**  **0.02** | **0.56**  **0.02** | 0.34  0.19 | **0.65**  **<0.01** | **0.51**  **0.04** | 0.29  0.26 | 0.35  0.17 | **0.59**  **0.01** | 0.39  0.12 | 1  - | 0.41  1 | **0.81**  **0.02** | 0.45  1 | 0.43  1 | -0.34  1 |
| 16. Instructions | rho  p | **0.57**  **0.02** | -0.07  0.78 | **0.60**  **0.01** | -0.08  0.75 | 0.43  0.08 | **0.58**  **0.01** | 0.29  0.26 | 0.15  0.57 | **0.55**  **0.02** | **0.70**  **<0.01** | **0.64**  **0.01** | 0.44  0.08 | **0.81**  **<0.01** | **0.72**  **<0.01** | 0.41  0.11 | 1  - | 0.32  1 | 0.62  1 | 0.57  1 | -0.38  1 |
| 17. Lang_matching | rho  p | 0  1 | -0.10  0.70 | 0.05  0.85 | -0.43  0.08 | **0.58**  **0.01** | **0.48**  **0.05** | **0.52**  **0.03** | 0.29  026 | 0.47  0.06 | 0.46  0.06 | 0.20  0.43 | 0.27  0.30 | 0.37  0.14 | 0.25  0.33 | **0.81**  **<0.01** | 0.32  0.21 | 1  - | 0.21  1 | 0.30  1 | -0.28  1 |
| 18. Figure_copy | rho  p | **0.56**  **0.02** | 0.41  0.11 | 0.14  0.58 | -0.01  0.96 | 0.33  0.20 | **0.53**  **0.03** | 0.07  0.80 | 0.32  0.21 | 0.26  0.31 | **0.83**  **<0.01** | **0.75**  **<0.01** | 0.36  0.15 | **0.70**  **<0.01** | **0.73**  **<0.01** | 0.45  0.07 | **0.62**  **0.01** | 0.21  0.43 | 1  - | **0.83**  **0.01** | -0.30  1 |
| 19. Figure_recall | rho  p | 0.48  0.05 | 0.39  0.13 | 0.10  0.69 | -0.07  0.80 | 0.38  0.14 | **0.57**  **0.02** | 0.02  0.93 | 0.34  0.19 | 0.39  0.12 | **0.89**  **<0.01** | **0.75**  **<0.01** | 0.44  0.08 | **0.61**  **0.01** | **0.72**  **<0.01** | 0.43  0.09 | **0.57**  **0.02** | 0.30  0.24 | **0.83**  **<0.01** | 1  - | -0.53  1 |
| 20. LI | rho  p | -0.28  0.27 | -0.24  0.36 | -0.16  0.53 | 0.26  0.31 | -0.20  0.43 | -0.20  0.43 | -0.08  0.75 | 0.14  0.59 | **-0.60**  **0.01** | **-0.49**  **0.05** | -0.04  0.87 | **-**0.47  0.05 | -0.35  0.17 | -0.18  0.49 | -0.34  0.19 | -0.38  0.13 | -0.28  0.27 | -0.30  0.24 | **-0.53**  **0.03** | 1  - |

Correlations above the diagonal are adjusted for multiple comparisons using Holm’s method. Bold values are significant (p < 0.05). Age_SO, age of seizures onset; Szr_freq, seizures frequency; FSIQ, full-scale IQ; PS_Index, processing speed index; VC_Index, verbal comprehension index; PR_Index, perceptual reasoning index; WM_Index, working memory index; Attntn_fig, figures test; DS_backwards, backwards digits span; AV, auditory list; LI, laterality index.

| **Table S4**  Cognitive performance across different numbers of concurrent antiseizure medications | | | |
| --- | --- | --- | --- |
|  | 2 drugs | 3 drugs | 4 drugs |
| FSIQ | 74.20 (11.48) | 60.11 (11.50) | 69.33 (17.50) |
| PS_Index | 85.40 (17.33) | 67.78 (11.70) | 78.00 (10.00) |
| VC_Index | 76.60 (12.03) | 65.44 (16.30) | 67.00 (20.78) |
| PR_Index | 78.80 (10.03) | 69.44 (8.17) | 89.00 (27.87) |
| WM_Index | 79.00 (13.30) | 65.11 (9.45) | 65.00 (13.75) |
| Attention_figures | 28.60 (8.96) | 19.33 (16.09) | 23.67 (8.62) |
| Attention_letters | 25.20 (9.07) | 14.89 (12.50) | 18.00 (14.93) |
| Digit_span_backwards | 4.20 (0.84) | 3.33 (2.18) | 3.33 (2.31) |
| Memory_AV_coding | 31.00 (7.87) | 23.67 (7.70) | 15.00 (3.61) |
| Memory_AV_recall | 7.20 (2.59) | 5.56 (2.51) | 4.33 (4.04) |
| Naming | 11.20 (4.09) | 10.33 (2.12) | 9.33 (5.51) |
| Instructions | 8.70 (0.67) | 7.33 (1.58) | 6.67 (0.58) |
| Language_matching | 14.80 (0.45) | 12.50 (4.03) | 14.33 (1.15) |
| Figure_copy | 11.00 (3.16) | 9.33 (5.43) | 7.33 (4.51) |
| Figure_recall | 8.20 (3.35) | 5.89 (3.89) | 5.67 (3.79) |

FSIQ, full-scale IQ; PS_Index, processing speed index; VC_Index, verbal comprehension index; PR_Index, perceptual reasoning index; WM_Index, working memory index; Attntn_fig, figures test; DS_backwards, backwards digits span; AV, auditory list
